# Chicago Veterinary Society

**Published:** 1899-06

**Authors:** 


### CHICAGO VETERINARY SOCIETY.

On Thursday, February 9th, President Robertson called the regular monthly meeting to order, and Drs. McCoy, Merillat, Howe, Campbell, E. L. Quitman, Clancy, and President Robertson showed their courage by braving the bitterness of 20° below zero, and were well repaid, even though they were compelled to endure the hardship of a meeting-room as cold as the street corner.

A motion by Dr. Quitman for the suspension of the roll-call and regular order of business was supported by Dr. Campbell, and so ordered.

Some very interesting facts were presented by Dr. Merillat concerning the ups and downs of the procedure of a bill before the State Legislature; the course it must travel back and forth; how it is lost and found, hurried and balked, and often killed by amendments and discussion, as well as the great necessity of a well-posted lobbyist, who should keep after it until it is signed or killed. He then presented a bill for the society's consideration for State regulation of the practice of veterinary medicine, and felt quite confident that it would become a law if pushed when presented to the Legislature for action. After discussing its merits for some time Dr. Quitman moved that the society approve of it and exert every effort for its advancement, which was supported by Dr. McCoy and so ordered. There being no further business for consideration, the meeting was adjourned.

The regular monthly meeting was called to order Thursday, March 9th, President Robertson presiding, and the following members were present: Drs. Allen, Campbell, Clancy, Dubia, Fish, Howe, McCoy, Nelson, Paxson, Robertson, and Walker. The roll-call was omitted, and the minutes of the previous meeting were read and approved.

President Robertson read some of the scores of letters received by members from Senators, Representatives, and citizens who complied with requests made of them to exercise all possible influence for the advancement of an amendment to the army reorganization bill, as petitioned by Dr. D. E. Salmon, chairman of the Committee on Army Legislation of the A. V. M. A., and speaking for this city he expressed the opinion that veterinarians in this locality had done their duty in the matter, as evidenced by the numerous letters before him, and felt that the promoters had earned the praise and gratitude of the whole profession for the labor achieved.

On a motion presented by Dr. Walker it was resolved that the Legislative Committee should communicate with the different candidates for the office of mayor and ascertain their intention in regard to the appointment of a qualified veterinarian to attend to the needs of the city horses.

On motion of Dr. Fish, supported by Dr. Dubia, it was resolved that the Secretary should forward excerpts of the foregoing resolution to the city press for publication.

Dr. R. G. Walker's paper proved very interesting, and brought forth a spirited discussion, with the following participants: Drs. H. D. Paxson, F. Allen, W. E. Howe, J. G. Fish, H. Busman, L. Campbell, R. Dubia, F. McCoy, and President Robertson.

JOSEPH B. CLANCY,
Secretary.

### DR. ROBERT G. WALKER'S PAPER.

*Gill-fliet.* (*Laceration of the Perineum*). The injury is a laceration of the space between the anus and the genital organs, sometimes including the sphincter ani. In this case feces pass from the rectum into the vagina, the voiding of feces being to some extent difficult and more or less incomplete and offensive. Many mares in this condition have been used as brood-mares with good results. Cases are reported by members of the profession to have made complete recovery after surgical treatment. The so-called gill-fliet is unsound.

*Paralysis of Sphincter Ani, of Tail, and Paralysis of Penis.* If such conditions exist at the time of examination, unsound. Paralysis of sphincter ani and tail is generally the result of a blow or fall on the rump, which sometimes causes a fracture of the sacrum and injury to the nerves supplying the tail and rectum, including the muscles of that region. You can have paralysis of the tail without paralysis of the sphincter ani, and, again, both conditions at the same time. I have had many cases of paralysis of the tail that have made successful recovery. The same cases came early under my care—say a few days after an accident, or as soon as the condition was noticed by the owner or attendant. I have seen horses in the hands of horse-dealers that could neither raise nor switch their tails. How long they had been that way I could not say, but have no idea that any treatment would be successful. Veterinarians examining horses for soundness should not omit examination of the tail.

*Curvature of Spine.* Roach, or high back, the opposite of low back, is frequently produced by the animal being put to draw and back heavy loads when very young; but many cases are not the result of work, as the same conditions have been noticed long before the animal ever had a harness on. When it occurs to a moderate extent only it does not impede the animal in his work, and he is therefore sound. When it is a positive disfigurement to the horse it is said to be a blemish. When the back is weakened or the horse is thereby impeded in his work he is unsound.

*Melanosis.* Common in gray and white horses on the black parts of the skin at the root of the tail, around the anus, vulva, udder, sheath, eyelids, and lips. Some authors recommend removal with the knife; others recommend no treatment. I have seen cases when members of the profession have operated where very bad results followed. In all cases that have come to my notice where I have been able to keep trace of the animal, the older the animal the more aggravated the case; therefore I would not hesitate on examinaton to pronounce the animal unsound.

*Enchondroma of Cariniform Cartilage, unsound; Broken Ribs,* on examination, unsound. I have treated several cases, which have all made speedy recovery, leaving no bad effects. I have always applied the cold pack or wet blanket, keeping the animal on a laxative diet, noting the pulse and temperature.

*Sitfasts.* It would require to be a very much worse case of sitfast than any which has yet come across my path to be pronounced unsound, as in all cases I have had of sitfast speedy recovery followed very little treatment; but I would advise that clients be informed, as bad results might happen if care be not taken.

*Lumbar Anchylosis.* Unsound.

*Phimosis.* A morbid condition of the prepuce or sheath, which, from contraction of the orifice, prevents the drawing or exit of the penis (Percivall). Blows, kicks, contusions, wounds, and abscesses within the sheath may all be set down as occasional causes on examination, and, finding such conditions, unsound (Williams).

*Paraphimosis.* The penis is protruded in paraphimosis, and cannot be withdrawn within the sheath. It may arise from injury or from debilitating disease, as in purpura. Frost-bite is very often the cause. I have had several cases the result of frost-bite. The first case that came under my notice was in the hospital of a city veterinaran, and lasted for several weeks. Amputation was resorted to, but with very bad results. The last case I saw had been under the care of a city veterinarian, who amputated a portion of the penis and returned the animal to the owner, who was a horse-dealer. The horse-dealer disposed of the animal to a client of mine, and three days after purchase I was sent for and informed that the horse could not make water. I was satisfied by the symptoms that the owner was right, and so got hold of the penis with the intention of using the catheter, but found amputation of a portion had been performed. I so informed the owner, but it was impossible to pass the catheter. I recommended the owner to try and return the horse to the dealer, so an arrangement was made that the dealer take back the horse, which he did within a few hours afterward; but the horse died in the dealer's stable the next morning. I think that very unsatisfactory results follow the amputation of a portion of the penis, and, if successful, owing to the length of time it takes for the animal to recover the owner would object to pay the bill; and probably the owner might have many lawsuits on his hands on account of the animal urinating on the sidewalk in place of the street. The cases caused by frost-bite I have treated have caused me very little trouble, and in no instance have I had a return of the trouble. I have always used an ointment of boric acid, tincture of benzoin, and cosmoline, covering the penis entirely with the ointment and cotton batting, using a support, tightening the support on the second and third days, redressing again, and repeating support two days more; then pushing the penis as far up into the sheath as possible, using the support again, and at the end of two days again. I have always been able to get all of the penis up into the sheath, still using the support and placing cotton up into the sheath, and in a very short time the animal had made a successful recovery.

*Paralysis of Penis*, if existing at time of examination, certainly unsound. I have had one case of paralysis of penis treated just in the same manner as frost-bite. The animal was in my hospital for thirty days, when he was able to go to regular work. The following July the horse was returned to my hospital in the same condition, and treated as before, working in thirty days, and it is now seven years since, and the animal, when I saw him last, about four months ago, was doing his regular work, and no paralysis of the penis existed. I have, in all cases treated, kept the animal on laxative food, with nux vomica.

*Mammitis.* Inflammation of the mammary gland; unsound. I have been very successful in mammitis after applying camphor and lard, but have had no good results from hot fomentations, poultices, or belladonna ointment.

*Orchitis.* Inflammation of testicles; unsound.

*Cancer of Penis.* Unsound.

*Hydrocele.* Dropsy of the scrotum; unsound.

*Hernia.* Unsound.

*Scirrhus Cora.* Unsound.

*Discussion Following Dr. R. G. Walker's Paper.*

Dr. Paxson: Dr. Walker mentions a case of paraphimosis due to paralysis, and recommends simply a suspensory, which has no therapeutic value. I think an amputation in some cases would have been safe.

Dr. Walker: The horse I referred to, that was operated upon and died, was of no great value. I was called to it when I knew that the horse could not live. I made an examination, but could not find any opening; and no water, but matter, came out of the penis. If it had been a very valuable animal I probably would have had to operate again. I thought it was hopeless anyway when the pus came out in this way; therefore I thought it best not to recommend an operation. Every case that I have seen operated upon was very unsatisfactory. As to the one I treated with a support, I had so much success with similar treatment before that I considered it to be the best.

Dr. Allen: I cannot agree with Dr. Walker as far as amputation of the penis is concerned. I have had two or three cases that I operated upon, and they are all right, though I do not claim to be a better operator than anybody else.

Dr. Howe: Dr. Walker, I would like to ask, in cases where you amputated, how much did you remove from the penis?

Dr. Walker: From four to six inches.

Dr. Howe: I operated on one, but do not know the result.

Dr. Robertson: I had one case where a horse's penis was very badly swollen for some time, and when brought to me the top was ready to slough off. I amputated the head and seared it. I have had considerable hemorrhage, but searing stopped it. The horse seems to be working all right ever since. I do not see why there should be any trouble following amputation of the penis. In my opinion, this case of paralysis that the doctor mentions, that resulted favorably by treating it with ointments, was because the support and the ointment lessen the inflammation considerably. I have seen several cases where support, without fomentations, was all right. I know of another case, treated by Dr. Hughes, where cold fomentations had a good effect. Where we have complete paralysis of the penis it is rather hard to get the penis back again.

Dr. Clancy: How do you operate, Dr. Allen?

Dr. Allen: Put in an old catheter, make a circular incision with a knife, and then use the écraseur..

Dr. Busman: I had two cases I operated upon, and both with good results. One was where the penis was frozen during the winter, and the other was the result of a scirrhus cord. The one with the frozen penis was brought to me in the spring. I operated on both with good results. Mode of procedure: I left about two inches of the urethra; stitched it back, and the remainder I cut off with an écraseur, and left the catheter in for about three days.

Dr. Campbell: Did not the stitch come out?

Dr. Busman: I do not think it did; anyway, not to my knowledge.

Dr. Robertson: I believe the essayist said that he pronounces a case of sitfast as sound. Does he do so in every instance?

Dr. Walker: I had some cases of sitfast that were in very bad condition; still, I never had a case that was incurable. A dealer never tries to sell a horse with sitfast that has gone so bad that it could not be cured if properly attended to. Anyway, I recommend the client to give the horse proper attention.

Dr. Campbell: What is the best way of curing sitfast?

Dr. Walker: Simply cutting it out.

Dr. Dubia: I do not think that anybody would be justified in passing an animal as sound if it cost the owner some money to treat it afterward.

Dr. Walker: I usually tell the purchaser about it.

Dr. Robertson: In regard to melanotic tumors, do you say that removing them is not a successful operation?

Dr. Walker: In many cases it is not.

Dr. Robertson: I had one case in a gray mare. The size of the tumor was nearly as large as a man's head, weighing about eight pounds, which I removed successfully, and had very little hemorrhage.

Dr. Campbell: Did any one of the members see a case of melanotic tumors in any other but a gray horse?

Dr. Clancy: I have seen some in a chestnut, but they were very small.

Dr. Allen: The essayist states that scirrhus cord is unsound; how can you differentiate a case of very small scirrhus cord and a case of recent castration?

Dr. Walker: I would make a very close examination, and if the animal was but recently castrated, I would call the attention of my customer to it.

Dr. Allen: Can a scirrhus cord be treated with iodide of potash successfully?

Dr. Walker: Yes.

Dr. Allen: Would you treat a very bad case with iodide of potash; say one weighing seven or eight pounds.

Dr. Walker: I might. I have never removed one myself, but I understand that bad cases were treated successfully with iodide of potash.

Dr. Robertson: I had a case of that kind in a horse that had quite a discharge from the scrotum. I inserted a probe, opened it up a little, so as to have room for the syringe, and treated the horse internally with iodide of potash, and in two weeks the horse was all right.

